# Cryptic introgression: evidence that selection and plasticity mask the full phenotypic potential of domesticated Atlantic salmon in the wild

**DOI:** 10.1038/s41598-018-32467-2

**Published:** 2018-09-18

**Authors:** Kevin A. Glover, Monica F. Solberg, Francois Besnier, Øystein Skaala

**Affiliations:** 1 Institute of Marine Research, P.O. Box 1870, N-5817 Bergen, Norway; 2 University of Bergen, Department of Biology, P.O. Box 7803, N-5020 Bergen, Norway

## Abstract

Domesticated Atlantic salmon grow much faster than wild salmon when reared together in fish tanks under farming conditions (size ratios typically 1:2–3). In contrast, domesticated salmon only display marginally higher growth than wild salmon when reared together in rivers (size ratios typically 1:1–1.2). This begs the question why? Is this a difference in the plastic response driven by divergent energy budgets between the two environments, or is it a result of selection, whereby domesticated salmon that display the greatest growth-potential are those at greatest risk of mortality in the wild? We reared domesticated, hybrid and wild salmon in a river until they smoltified at age 2 or 4, and thereafter in fish tanks for a further 2 years. In the river, there was no difference in the mean size between the groups. In contrast, after being transferred from the river to fish tanks, the domesticated salmon significantly outgrew the wild salmon (maximum size ratio of ~1:1.8). This demonstrates that selection alone cannot be responsible for the lack of growth differences observed between domesticated and wild salmon in rivers. Nevertheless, the final size ratios observed after rearing in tanks were lower than expected in that environment, thus suggesting that plasticity, as for selection, cannot be the sole mechanism. We therefore conclude that a combination of energy-budget plasticity, and selection via growth-potential mortality, cause the differences in growth reaction norms between domesticated and wild salmon across these contrasting environments. Our results imply that if phenotypic changes are not observed in wild populations following introgression of domesticated conspecifics, it does not mean that functional genetic changes have not occurred in the admixed population. Clearly, under the right environmental conditions, the underlying genetic changes will manifest themselves in the phenotype.

## Introduction

The domestication of Atlantic salmon (*Salmo salar* L.) was initiated in the early 1970’s when wild fish were collected from a selection of Norwegian rivers and thereafter used as broodstock in breeding programs^1,2^. For each generation that passed, fish were exposed to directional selection for an increasing number of economically important traits, such as rapid growth, delayed maturation, desirable flesh characteristics and increased disease resistance^2,3^. Genetically improved strains of Atlantic salmon for commercial aquaculture have subsequently been established in most of the major salmon producing countries^4–7^, and this species is regarded as one of the most domesticated fish reared for food^8^.

Genetic differences in the expression of a wide range of phenotypic traits now exist between domesticated and wild Atlantic salmon^9^. However, the trait displaying the largest and most consistent difference between domesticated and wild salmon is growth, typically measured as size at age, under controlled farming conditions^10–13^. This stands to reason given that growth displays a high heritability in salmon^3,14^, and has represented the most important target of selection. The most recent studies typically reveal between 2 to 3-fold differences in size at age between domesticated and wild fish when reared together in fish tanks under farming conditions^11–13,15^, although up to 5-fold differences have been documented^15^. Multiple quantitative trait loci associated with growth have been reported in Atlantic salmon^16–18^, perhaps not surprisingly, as this trait is regarded to be under polygenic control^19^. Variation in this trait is primarily, although not exclusively^20^, explained by an additive genetic model. This is further evidenced by the fact that F1 hybrids typically display intermediate growth rates between their fast growing domesticated and slow growing wild parents^13,15,21,22^.

As for other species of fish, growth is highly plastic in salmon^11,15,22,23^. Significantly, there are differences in reaction norms between domesticated and wild salmon, and in the natural environment, growth of domesticated salmon, measured as size at age, is only marginally higher than for wild salmon^17,24–27^. This stands in stark contrast to the very large differences observed in growth between domesticated and wild salmon when reared together in fish tanks^10–13,15^, and begs the question, why? Is this driven by a difference in energy budgets between the two environments? For example, as rivers display a limited production capacity^28–30^, could food be limiting in the natural environment, thus not providing domesticated salmon with enough energy to utilize their high genetic growth-potential and outgrow wild salmon? Also, are domesticated salmon less effective at catching prey in the wild, and do they expend more energy in their search for them? Or alternatively, could individual domesticated salmon displaying the highest growth-potential be more susceptible to predation in the wild than individual domesticated fish displaying lower growth-potential? Such a selection mechanism could result in more similar growth, recorded as size at age, among surviving domesticated salmon and wild salmon in the natural environment. Although some of the selection during freshwater is density-dependent^28,29^, earlier studies have suggested that deviating growth-potential mortality may influence the growth relationship between domesticated and wild salmon^15^, and that growth-potential may be negatively correlated with survival in the wild^31–33^. Survival from egg to smolt is typically ~1–3% for Atlantic salmon in the wild^24,34–36^, and the offspring of domesticated salmon display higher mortality rates than the offspring of wild salmon in the natural environment^24,27,34,37^. Thus, natural selection has significant potential to select against sub-optimal phenotypes, such as domesticated fish displaying a high genetic growth capacity. In turn, this could result in more similar size distributions between fish of domestic and wild pedigree in the natural environment.

Using two complimentary approaches, the present study was designed to investigate why domesticated and wild salmon display deviating growth distributions across the contrasting natural and farming environments. In the first approach, we studied growth of domesticated, F1 hybrid and wild Atlantic salmon that lived the first 2–4 years of their lives in a natural river system, that were thereafter transferred to fish tanks and reared on high-energy pellets for a further 2 years. Our reasoning was such: if high growth-potential and/or size-selective mortality (hereon collectively referred to as “growth-potential” mortality) represents the primary mechanism preventing size differences developing between the groups in the wild, then the growth reaction norms of survivors from the wild should be similar when transferred to a high energy commercial diet in fish tanks. If however, this primarily represents a genetic difference in the plastic response to the deviating energy-budgets between the environments, then the domesticated salmon would be expected to out-grow the wild salmon when transferred to fish tanks and provided a high energy diet.

In the second approach, and to compliment the main experiment, we implemented a weight-dependent mortality function to simulate selection via growth-potential mortality on a data set documenting growth of wild, domesticated and hybrid salmon smolts in fish tanks. We did this to investigate whether it was possible to create overlapping weight distributions of these genetic groups, similar to the weigh distributions of these groups observed in a river^24^, by a size-dependent mortality function alone. The mortality rates required to create overlapping weight distributions between domesticated and wild salmon were thereafter compared to observed mortality rates of these groups in the wild.

## Results

### Numbers of fish sampled in the river and reared thereafter in tanks

In the period 14–22 May 2012, 360 seaward-migrating salmon smolts that had survived in the river Guddal since being planted out as eyed eggs in 2008 and 2010, were sampled on the downstream fish trap. These survivors were thereafter transferred to fish tanks at the Matre fish farm for continued rearing. DNA parentage testing revealed that the majority of the 360 fish gathered from the trap were 4 year-old smolts from the 2008 cohort, and of wild pedigree (268/10, 14/9 and 56/3 wild, hybrid and domesticated individuals, from the 2008/2010 cohorts respectively). Nevertheless, all three genetic groups, and both smolt age groups, were represented in the total material collected. These data are attached (Supplementary File 1).

### Growth of fish sampled in the river and reared thereafter in tanks

**Figure 1:**
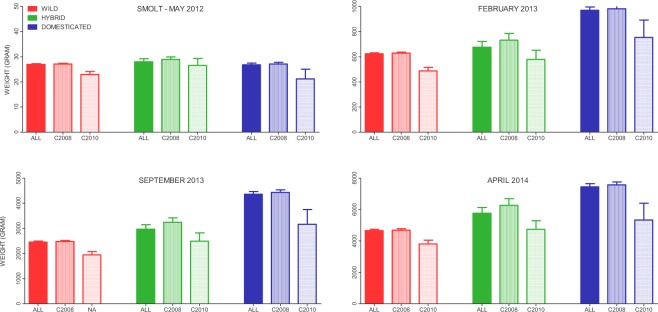
Mean size (g) of wild, hybrid and domesticated salmon as measured at timepoint 1 (May 2012, sampled in the river, age 2–4 years), and timepoints 2–4 (February 2013-April 2014, sampled in fish tanks, age 3–6 years). Error bars show the standard error.

**Figure 2:**
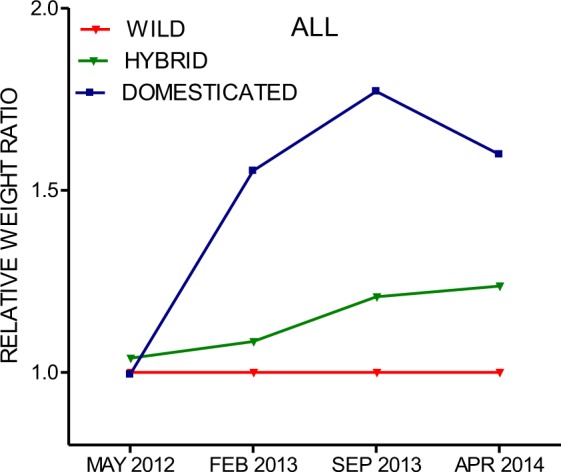
Size ratio among wild, hybrid and domesticated salmon sampled at timepoint 1 (May 2012, sampled in the river, age 2–4 years), and timepoints 3–4 (February 2013–April 2014, sampled in fish tanks, age 3–6 years).

No significant difference in smolt size was observed among salmon of domesticated, hybrid and wild pedigree when measured on the downstream fish migration trap in the river at timepoint 1 (Fig. 1, Table 1). This gave an overall size ratio of ~1:1 at the smolt stage in the wild (Fig. 2). However, after 10, 17 and 24 months of rearing in tanks (timepoints 2, 3 and 4 respectively), significant differences in fish size were detected among salmon of domesticated, hybrid and wild pedigree (Fig. 1, Table 1) (Strain^x^time interaction: F_6,957_ = 65.6, P < 0.001, Strain: F_2,21_ = 19.7, P < 0.001, Time: F_3,957_ = 29 332, P < 0.001). The largest difference between these groups was detected after 17 months of tank rearing, resulting in a maximum size ratio of 1:1.8 between wild and domesticated salmon (Fig. 2).

**Table 1:** Statistical results for growth (size) and condition factor of domesticated, hybrid and wild salmon upon sampling at timepoint 1 (aged 2–4 years, sampled in river) and timepoints 2–4 (aged 3–6 years, sampled in fish tanks).

	Domesticated				Hybrid				Wild			
Timepoint	1	2	3	4	1	2	3	4	1	2	3	4
Weight (g)	*27.0*	*979.9*	*4356.5*	*7450.0*	*28.1*	*676.4*	*2968.2*	*5660.0*	*27.2*	*623.6*	*2459.2*	*4659.7*
K	0.83	1.21	1.40	1.29	0.86	1.11	1.27	1.27	0.85	1.14	1.27	1.23
Domesticated	—	—	—	—	**0.41**	<0.001	<0.001	**0.38**	**0.21**	<0.001	<0.001	<0.001
Hybrid	***0.23***	*0.001*	*0.002*	*0.03*	—	—	—	—	**1.00**	**0.16**	**0.93**	**0.10**
Wild	***0.77***	<*0.001*	<*0.001*	<*0.001*	***0.13***	***0.09***	*0.003*	*0.001*	—	—	—	—

Upper and lower triangle shows the P-value of the pairwise comparisons of the condition factor (K) and the body weight (italic), respectively, of domesticated, hybrid and wild salmon at the different time points. Significant results are marked in bold.

A significant difference in mean size was observed between fish from the two cohorts, with older smolts being larger (cohort/smolt age: F_1,26_ = 8.3, P = 0.008) (Fig. 1). This difference in weight between cohorts were consistent for all three genetic groups (Strain^x^cohort interaction: F_2,26_ = 0.48, P = 0.6), and across all time points (Cohort^x^Time interaction: F_3,26_ = 0.48, P = 0.6). Thus, the fish that smoltified at an older age, were consistently larger throughout the whole study, within their respective genetic groups.

### Mortality and condition factor

After 2 months of being reared in fish tanks on a combined diet of insect larvae (bloodworms, *Chironomidae)* and pellets, daily observations indicated that all fish were weaned onto pellets. This observation was supported by the low mortality observed during this and subsequent periods. During this transition period, and up until the next sample was taken (timepoint 2, ten months after transfer fish tanks), overall mortality was 8.33%. This corresponded to 8.63% (N = 24), 4.35% (N = 1) and 8.47% (N = 5) of the wild, hybrid and domesticated individuals respectively.

**Figure 3:**
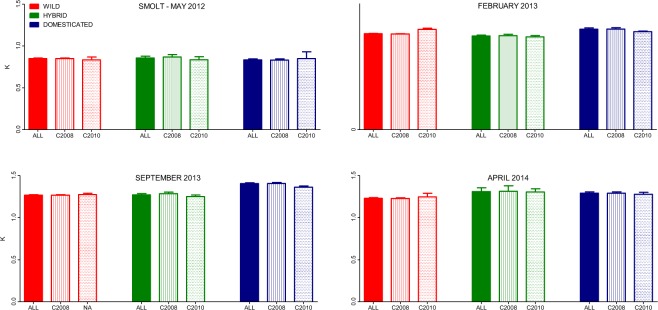
Mean condition factor of wild, hybrid and domesticated salmon sampled at timepoint 1 (May 2012, sampled in the river, age 2–4 years), and timepoints 2–4 (February 2013-April 2014, sampled in fish tanks, age 3–6 years). Error bars show the standard error.

No significant difference in condition factor were observed among salmon of wild, hybrid and domesticated pedigree sampled on the fish trap at timepoint 1 (Fig. 3, Table 1) and all fish displayed a significant increase in condition factor after a period of rearing in the fish tanks (Fig. 3, Table 1). While hybrid and wild salmon displayed similar condition factors throughout the tank rearing stage of the experiment, domesticated salmon displayed an increasing K (Strain^x^time interaction: F_6,962_ = 13.4, P < 0.001, Strain: F_2,20_ = 12.3, P = 0.003, Time: F_3,963_ = 775.6, P < 0.001, Table 1).

No significant difference in condition factor was observed between fish from the two cohorts at any time point, in any of the genetic groups (Strain^x^cohort interaction: F_2,43_ = 0.3, P = 0.7, Time^x^Cohort interaction: F_3,965_ = 1.02, P = 0.4, Cohort: F_1,41_ = 0.02, P = 0.9).

### Modelling size selective mortality

We modelled size-selective mortality, as a proxy for growth-potential mortality, to investigate whether it was possible to create overlapping size distributions using selection alone. This was achieved using a simulated data set consisting of 10^7^ wild, hybrid and domesticated individuals created using the mean and the standard deviation of the genetic groups’ weight in a previously published data set documenting growth of salmon from multiple strains in fish tanks^38^ (Supplementary File 2).

**Figure 4:**
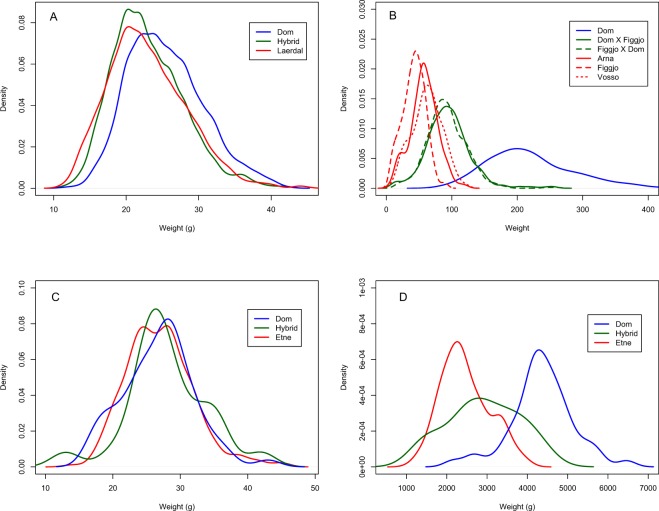
Weight distributions of domesticated, hybrid and wild salmon from data sets collected in the wild, and from fish tanks: (**A**) Smolts sampled in the river Guddal from a previously published study of survival and growth^24^, (**B**) Smolts sampled from multiple strains in fish tanks that had been reared in that environment from hatching onwards^38^ and that were used for the modelling simulations in this work, (**C**) Smolts sampled in the river Guddal in 2012 that form the experimental work in the present study (N = 360), (**D**) Smolts transferred from the river Guddal to the fish farm and after ~17 months of rearing in fish tanks (N = 321). Dom = domesticated strain, Dom × Figgjo and Figgjo × Dom = hybrid variants, while Lærdal, Figgjo, Arna, Vosso, and Etne = wild populations.

In a previous study where growth of wild, hybrid and domesticated salmon was investigated in the river Guddal^24^, observed smolt weights were highly overlapping between the genetic groups (22.9 ± 5.3, 23.0 ± 4.9 and 25.4. ± 5.1 g respectively, Fig. 4A). In contrast, divergent weight distributions were observed in the data set documenting growth of multiple wild, hybrid and domesticated salmon smolts reared in fish tanks at the Matre fish farm (54.9 ± 22.5, 93.6 ± 32.5 and 217.6 ± 65.1 g respectively, Fig. 4B)^38^. This data set was used for the growth-potential mortality simulation here. We set the mean observed weight of the wild fish in the tanks (54.9 g) as the optimum for survival, based upon the assumption that wild fish growth represents the optimum resulting from natural selection. Thereafter, the probability of mortality in all three genetic groups was modelled as a function of each individual fish’s weight-deviation from the optimum. The results of this simulation demonstrated that in order to create overlapping weight distributions between salmon of wild, hybrid and domesticated origin reared under farming conditions, the differential mortality rates of the three genetic groups needed to be far greater than observed in the wild (Table 2 vs.^24,27,34,37^). Thus, this simulation, which was used to supplement the main growth study, suggests that growth-potential or size-selective mortality alone cannot explain why domesticated salmon do not outgrow wild salmon in the natural environment.

**Table 2:** Modelled mortality rates and the resulting weight distributions of domesticated, hybrid and wild salmon.

x	Weight	Resulting mortality			Weight (gram ± SD)		
	optimum	Domesticated	Hybrid	Wild	Domesticated	Hybrid	Wild
		%	%	%	Mean	Mean	Mean
0.01	mean(Wild)	85.1	80.1	80	198 ± 58	93 ± 32	55 ± 22
0.025	mean(Wild)	96.0	82.0	80.2	144 ± 51	88 ± 29	54 ± 22
0.05	mean(Wild)	98.9	88.1	81.3	96 ± 36	76 ± 23	54 ± 19
0.075	mean(Wild)	99.5	91.7	83.8	76 ± 25	68 ± 19	54 ± 16
0.1	mean(Wild)	99.8	93.7	86.2	67 ± 19	64 ± 16	54 ± 14

The initial data set used for this modelling was smolt weights of domesticated, hybrid and wild salmon from tank-reared salmon (Fig. 4B). The average weight of the wild salmon was set as the optimal for survival. x represents the parameter changing the strength of weight-dependent mortality function, implemented as a proxy to simulate growth-potential mortality.

## Discussion

This is the first study to investigate growth of domesticated, hybrid and wild salmon in the natural environment, and thereafter in fish tanks. After 2–4 years in the river, no difference in size was observed among these genetically distinct groups (Figs 1, 2, 4C). However, once these fish were transferred from the river to tanks, and fed a high-energy pellet diet, the genetic growth potential of the domesticated salmon was suddenly unleashed, resulting in highly significant size differences (Figs 1, 2, 4D). This demonstrates that selection alone cannot be responsible for the lack of growth differences observed between domesticated and wild salmon in rivers. Nevertheless, the largest size ratios observed here after the fish had been transferred to and reared in fish tanks (1:1.8), were lower than typically reported between domesticated and wild fish reared in that environment (typically 1:2–1:3, albeit with exceptions)^10–12,15,39^. Furthermore, the differences were much lower than observed in a study conducted in fish tanks (1:2.9)^11^ that used part of the same genetic material as in the present study. We therefore conclude that genetic differences between domesticated and wild salmon, in their plastic response to the divergent energy budgets between the river and fish tanks, cannot be the sole cause of the observed large differences in their norms of reaction across these contrasting environments. It therefore follows that a combination of selection and plasticity are responsible for the large differences in growth reaction norms between domesticated and wild salmon across the natural and farming environments.

The offspring of domesticated salmon display lower survival in the natural environment when compared to the offspring of wild salmon^24,27,34,37^. Studies have also indicated that the offspring of domesticated salmon are more naïve to predators than the offspring of wild salmon^40,41^, and that there is a trade-off between fast growing domesticated fish and survival^32,33^. Nevertheless, while growth-potential selection against the fastest growing domesticated fish could prevent size differences developing between domesticated and wild salmon in the natural environment, our results demonstrate that this cannot be the primary force at work. Clearly, the majority of the domesticated fish surviving for 2–4 years in the river still displayed a significantly higher growth potential than the wild fish, illustrated by the fact that they suddenly out-grew the wild salmon when transferred into fish tanks and onto a pellet diet (Fig. 4C vs. D). If selection via growth-potential mortality was the primary driver, we would not have expected to observe this. Other factors, potentially in combination with a degree of growth-potential selection, must therefore be responsible for the observed differences in growth reaction norms.

After modelling size-selective mortality on a data set documenting hatchery growth of smolts of domesticated, hybrid and wild origin, as a proxy for growth-potential selection, we were still not able to create overlapping weight distributions that are observed in the wild (Fig. 4A,C) at mortality rates similar to those observed for domesticated and wild salmon in rivers^24,27,34,37^. To create even close to overlapping weight distributions (Table 2), the morality rate of wild salmon had to be modelled far lower than what is observed for egg to smolt survival in the wild^24,34–36^, while the morality rate of domesticated salmon had to be modelled far higher than has been reported in the wild^24,34^. While the modelling exercise is arguably simplistic, it nevertheless supports the results from the main experimental part of this study.

It could be reasoned that the domesticated fish were able to outgrow the wild fish after being transferred from the river to fish tanks, due to genetic differences in their abilities to tackle this transition. While it is not possible to unequivocally exclude this possibility, we find it unlikely that it played a major role in our results for the following reasons. After being transferred to the fish tanks, fish of all genetic groups displayed very low mortality, substantial gains in growth (Figs 1, 2), and an increase in condition factor (Fig. 3), all of which demonstrate that fish of all genetic backgrounds managed the transition well. In addition, earlier studies have investigated whether domestication has led to adaptations to specific factors associated with farming conditions, such as; handling stress^11^, high rearing densities and social interactions^15,42^, feeding regimes^22^, and high-energy and pellet-based diets^39^. However, none of the above factors appear to inflate the relative growth differences between domesticated and wild salmon reared in fish tanks in farms.

Domestication of Atlantic salmon has resulted in a faster growing fish that displays increased consumption, metabolism and potentially feed conversion efficiency when presented with an excess and high energy diet in a protected environment such as a farm, as opposed to adaptations to the specific physical and social conditions of the farm per se^9^. Significantly, when growth has been investigated under farming and semi-natural conditions, both with restricted access to feed, differences between domesticated and wild salmon were less than under standard farming conditions with excess feed^15^. Thus, in the light of the results from the present study, it would seem highly likely that part of the difference in growth between domesticated and wild salmon in the natural habitat vs the fish farm is a difference in genetic based plasticity, mediated through the lack of available energy, or the accessibility of that energy, for the domesticated salmon to out-grow wild fish in the wild. This situation may also be exacerbated by domesticated salmon using more energy in the natural environment, as has been reported in growth-enhanced brown trout (*Salmo trutta* L.)^43^.

Over the past three to four decades, tens of millions of domesticated salmon have escaped into the wild^9,44^, and introgression of domesticated salmon has been documented in Norwegian^45–48^, Irish^49,50^, Scottish^51^, and Canadian populations^52,53^. All available evidence suggests that introgression of domesticated escapees represents a threat to the genetic integrity, abundance and long-term evolutionary viability of native populations^9^. Despite this, direct evidence of phenotypic, life-history or demographic changes in native populations where introgression of domesticated escapees has been documented, is sparse^9^. A recent multiple-river study demonstrated that fish hatched in the wild, but displaying domestication-admixture, had slightly higher average size at maturity than pure wild fish from the same river^54^. However, a modelling study indicated that at low to modest domestication introgression levels, clear differences in mean freshwater growth are unlikely to emerge in wild populations, in part due to selection against the offspring of domesticated salmon, and in part due to plasticity in growth^55^. In this context, results of the present study provide a salient reminder that even if clear phenotypic changes are not observed following introgression of domesticated escapees, it does not demonstrate that functional genetic changes have not occurred in the wild population. Changes in trait expression may occur in domestication-admixed populations over time, or under changing environmental conditions. To this end, our data clearly show that under the right conditions, the underlying genetic changes may manifest themselves in the phenotype – thus removing the sheep´s clothing, and unveiling the wolf.

## Materials and Methods

### Genetic material

The study is based upon domesticated, F1 hybrid and wild salmon. To produce the domesticated fish, we used the commercial Mowi strain. This had been in domestication for ≥10 generations at the time of stripping, and has previously been demonstrated to display several-fold higher growth rates, measured as size at age, than wild salmon under farming conditions^10,11,23^. To produce the wild fish, we used adult salmon from the river Etne. To ensure that these fish had been born in the wild, scales of broodfish were checked^56^. F1 hybrids between these two strains were produced by fertilizing eggs from a domesticated female with sperm from a wild male. In the autumn of 2007 and 2009, a total of 17 (7 wild, 2 hybrid and 8 domesticated) and 29 (10 wild, 9 hybrid and 10 domesticated) families were produced.

### Production of fish in the river Guddal

In the spring of 2008 and 2010, 69 800 (31 800 wild, 9 400 hybrid and 28 600 domesticated) and 106 000 eyed (34 800 wild, 32 000 hybrid and 39 200 domesticated) eggs were planted into the river Guddal in Hardanger, western Norway. This material forms part of an ongoing long-term study of fitness in the natural environment, following up from earlier work^24,57^. Consequently, details about group and family performance in the river are not presented as the fish used here only represent a small sub-set included in the main study.

The river Guddal is partly-fed by the Folgefonna glacier, so it is a summer-cold river. Consequently, salmon grow relatively slowly in this river, and the typical age for smolts is 3–4 years, although smolt ages of 1–5 years have been reported^24^. Survival from egg to smolt in this river is typically 1–3%^24^, and a native brown trout (*Salmo trutta* L.) population, that predates on emerging salmon juveniles exists^57^. This river is otherwise typical for a small Norwegian river located on the western part of Norway. After being planted out as eyed eggs, fish representing the above groups were left alone until they smoltified and exited the river.

In the spring of each year, smolts migrating out of the river Guddal are captured on a permanent trapping facility. In the period 14–22 May 2012, 360 fish, representing a random sub-set of the salmon smolts migrating out of the river that season were measured (weight, length, fin clip taken), and thereafter transferred to the Matre research facility (timepoint 1 in the results). The transferred sub-population consisted of a total of 278, 23 and 59 wild, hybrid and domesticated individuals, respectively, whereof 10, 3 and 9 individuals were 2-year-old smolts of the 2010 cohort, while the remaining where 4-year-old smolts of the 2008 cohort. In the sub-population, the 2008 and 2010 cohorts were represented by a total of 16 out of 17 (6 wild, 2 hybrid and 8 domesticated) and 10 out of 29 (4 wild, 4 hybrid and 2 domesticated) families initially planted in the river.

### Continued production of the fish in tanks at the Matre research facility

Upon arrival at Matre in late May 2012, fish from all genetic groups were randomly divided into two standard 1.5 m^2^ tanks, then weaned onto a commercial pellet diet by mixing insect larvae (bloodworm, *Chironomidae*) in the tanks together with pellets. After approximately 2 months, the fish had been weaned onto the commercial diet and supplementary feeding with bloodworm was ceased. During this transition period, and up until the next sample was taken (timepoint 2) ten months after transfer to Matre, overall mortality was only 8.33%. Fish were fed continuously in excess and held on a standard light regime for Bergen. The fish were reared in tanks for nearly two years. They were sampled in February 2013 (timepoint 2 in the results, N = 330). In this sample, fish were measured, fin clip taken and individually PIT tagged to provide subsequent identification. The fish were thereafter sampled in September 2013 (timepoint 3 in the results, N = 321) and April 2014 (timepoint 4 in the results, N = 310).

### DNA identification of fish

The fish included in this study were identified to their type (domesticated, F1 hybrid and wild) using DNA parentage testing. DNA analysis included amplification of six highly polymorphic microsatellite loci on an ABI sequencing machine located in the molecular genetics laboratory at the Institute of Marine Research, Bergen. DNA parentage testing, combined with an exclusion based assignment^58^, has been previously used in this laboratory to identify >20 000 fish to their families and groups of origin originating from both this river system^24,57^ and the Matre fish farm^11,15,23^.

DNA analysis was not only used to identify fish to their groups of origin, it was also used to connect individual fish and their biological data between sampling timepoint 1 and 2. Individual fish were identified to themselves based upon identical match in their multi-locus DNA profile. In the few cases where individuals shared an identical genetic profile to another fish, they were genotyped for a further five microsatellites to provide unambiguous individual identification. For full details regarding this procedure the reader is referred to previous work^59,60^.

### Statistics

All analyses were carried out using the R statistical software, version 3.4.3^61^. Linear mixed effect (LME) models were used to compare the growth trajectories, and the corresponding condition factor, of fish of wild, domesticated and hybrid genetic background. Two models were fitted using the *lmer* function in the lme4 package^62^. The full models tested for the effect of strain (S), i.e., wild/hybrid/domesticated, and cohort/smoltage (C), i.e., 2008 and 2010, and time (T), i.e., timepoints 1, 2, 3 and 4, as well as their interactions, upon log-weight (LogW) and condition factor (K). All fixed effects were coded as factors, with three, two and four levels, respectively. Due to repeated measurements of individuals in this study, their identity was included as a random effects (ID). Family (Fam) was also include as random effects, to control for variation between families. Due to the hierarchal structure of the data set, the random effects were nested. The full models:

$$LogW/K=\alpha +{\beta }_{1}S+{\beta }_{2}C+{\beta }_{3}T+{\beta }_{4}SC+{\beta }_{5}ST+{\beta }_{6}CT+{\beta }_{7}SCT+{b}_{ID(Fam)}+\varepsilon $$

where α is the intercept and ε is a random error. Model selection was performed backwards by the use of the *step* function in the lmerTest package^61^. Insignificant random effects were eliminated before the removal of insignificant fixed effects. Interaction terms were removed before the variables themselves:

$$LogW=\alpha +{\beta }_{1}S+{\beta }_{2}C+{\beta }_{3}T+{\beta }_{4}ST+{b}_{ID(Fam)}+\varepsilon $$

$$K=\,\alpha +{\beta }_{1}S+{\beta }_{2}T+{\beta }_{3}ST+{b}_{ID(Fam)}+\varepsilon $$

Each model’s fit was confirmed by examining the histogram of the model residuals and by plotting the model residuals against the fitted values. The potential autocorrelation of the time series in the models were investigated using the *acf* function.

### Simulation of growth-potential mortality

As a supplement to the main experiment, modelling was conducted to investigate whether selection alone, via growth-potential mortality, can create strongly overlapping size distributions of wild, hybrid and domesticated salmon that are typically observed in the wild. In order to achieve this, we implemented a size-selective mortality function, as a proxy for growth-potential mortality, on a data set documenting growth of three wild strains, a domesticated strain and reciprocal wild-domesticated hybrids in fish tanks under farming conditions where there has not been any selection^38^ (Supplementary File 2). For details about the production, rearing and sampling of these fish at Matre, see^38^.

Mortality was implemented in the above-mentioned data set as a function of the fish’s recorded smolt weight, using the observed mean weight of the wild salmon in the data set (54.9 g, Fig. 4B) as the optimum for survival (*W*_*opt*_). This was based upon the assumption that the mean weight of the wild fish represents the “optimum” growth-potential that has evolved in the wild. Thereafter, a morality function that gives the probability of mortality (*Pm*) as a function of the fish weight was implemented. The mortality function was then implemented on a simulated data set consisting of 10^7^ wild, hybrid and domesticated individuals created using the mean and the standard deviation of the genetic groups’ weight in the hatchery data set (54.9 ± 22.5, 93.6 ± 32.5 and 217.6 ± 65.1 g respectively, Fig. 4B). The absolute difference (*d*) between each individual (*W*_*i*_) and the optimum weight (*W*_*opt*_) was calculated as:

$$d=abs({W}_{i}-{W}_{opt})$$

while the normalized weight difference (*dn*) was calculated as:

$$dn=\frac{d-{\rm{mean}}(d)}{{\rm{sd}}(d)}$$

The probability of mortality (*Pm*) was then derived from a sigmoid function of *dn:*

$$Pm=\frac{1}{1+\exp (\,\,-\,{\rm{x}}.dn)}$$

where x is a parameter that changes the steepness of the curve, illustrating the strength of size selective mortality. *Pm* was at minimum when *W*_*i*_ = *W*_*opt*_, and the minimum mortality probability was set to 80% (i.e, *Pm* smaller than 0.8 was set to 0.8 a posteriori).

### Ethical statement

The experimental protocol (permit number 5296 (main experiment) and 5186 (data used in the modelling simulations)) was approved by the Norwegian Animal Research Authority (NARA). All welfare and use of experimental animals was performed in strict accordance with the Norwegian Animal Welfare Act. This included full anesthesia of fish using metacain (Finquel^®^ Vet, ScanVacc, Årnes, Norway), during all four samples referred to. In addition, all personnel involved in this experiment had undergone training approved by the Norwegian Food Safety Authority, which is mandatory for all personnel running experiments involving animals included in the Animal Welfare Act.

## Electronic supplementary material

Supplementary file 1

Supplementary file 2
